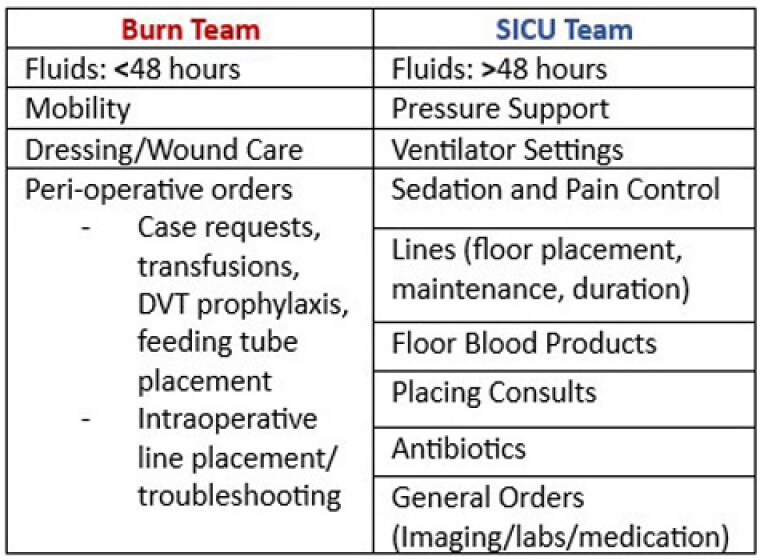# 664 Fanning down the Flames: Improving Burn and SICU Team Dynamics with Co-management Guidelines

**DOI:** 10.1093/jbcr/iraf019.293

**Published:** 2025-04-01

**Authors:** Kerilyn Godbe, Niaman Nazir, Stepheny Berry, Steve Eaton, Dhaval Bhavsar, Julia Slater

**Affiliations:** The University of Kansas Health System Burnett Burn Center; University of Kansas Medical Center; The University of Kansas Health System Burnett Burn Center; The University of Kansas Health System Burnett Burn Center; The University of Kansas Health System Burnett Burn Center; The University of Kansas Health System Burnett Burn Center

## Abstract

**Introduction:**

The main barriers to perioperative surgical teamwork are confusion in responsibilities, and prevailing misconceptions between teams. Our Burn and Surgical intensive care unit (SICU) teams noted these obstacles and sought to improve co-management of burn patients.

**Methods:**

Burn and SICU providers were surveyed to identify preferred management roles. Subsequently, two providers from each team created guidelines delineating management responsibilities. A five-point Likert scale survey was distributed pre- and six months post- guideline distribution to assess impact of intervention.

**Results:**

A total of 87.5% (14/16) of Burn (n=6) and SICU providers (n=8) participated in an initial survey on team role and dynamics, while 68.7% (11/16) participated in a post-survey. All providers (100%) agreed the SICU team should manage the ventilator, pressure support, and sedation, and the Burn team should manage wound care and mobility. There were discrepancies in fluid resuscitation management and general orders. All Burn providers desired to lead fluid resuscitation for the patient’s entire stay in contrast to 50% and 87.5% of SICU providers desiring management at < 48 hours and >48 hours, respectively. Although our sample size was not large enough to detect many statistically significant differences, the following trends were observed following guideline (Figure 1) implementation. Providers noted improved clarity in team roles (3.1, SD 1.0 vs 3.7, SD 1.2; p=0.10), more effective co-management of patients (3.6, SD 0.7 vs 4.0, SD 0.5; p=0.08), and improved inter-team communication (2.7, SD 0.9 vs 3.5, SD 0.9; p=0.06). After the intervention, more providers believed they were respected by the other team (3.2, SD 0.9 vs 3.6, SD 0.7; p=0.2), and there were less disagreements in management (1.9, SD 0.7 vs 2.9, SD 1.0; p=0.02). Additionally, 90.9% (10/11) providers agreed or strongly agreed these guidelines were helpful in delineating team roles, and 63.6% (7/11) of providers felt guidelines improved the Burn and SICU team relationship.

**Conclusions:**

Implementation of Burn/SICU team management guidelines may enhance team dynamic and provide team role clarification.

**Applicability of Research to Practice:**

Care coordination between surgical teams is integral to successfully manage critically ill patients. This is the first example of how guideline implementation could improve inter-team dynamics within the burn literature.

**Funding for the Study:**

N/A